# The Uniqueness of Tryptophan in Biology: Properties, Metabolism, Interactions and Localization in Proteins

**DOI:** 10.3390/ijms21228776

**Published:** 2020-11-20

**Authors:** Sailen Barik

**Affiliations:** 3780 Pelham Drive, Mobile, AL 36619, USA; barikfamily@gmail.com

**Keywords:** tryptophan, indole, virus, immunity, serotonin, kynurenine, codon

## Abstract

Tryptophan (Trp) holds a unique place in biology for a multitude of reasons. It is the largest of all twenty amino acids in the translational toolbox. Its side chain is indole, which is aromatic with a binuclear ring structure, whereas those of Phe, Tyr, and His are single-ring aromatics. In part due to these elaborate structural features, the biosynthetic pathway of Trp is the most complex and the most energy-consuming among all amino acids. Essential in the animal diet, Trp is also the least abundant amino acid in the cell, and one of the rarest in the proteome. In most eukaryotes, Trp is the only amino acid besides Met, which is coded for by a single codon, namely UGG. Due to the large and hydrophobic π-electron surface area, its aromatic side chain interacts with multiple other side chains in the protein, befitting its strategic locations in the protein structure. Finally, several Trp derivatives, namely tryptophylquinone, oxitriptan, serotonin, melatonin, and tryptophol, have specialized functions. Overall, Trp is a scarce and precious amino acid in the cell, such that nature uses it parsimoniously, for multiple but selective functions. Here, the various aspects of the uniqueness of Trp are presented in molecular terms.

## 1. Introduction

Tryptophan (Trp, W) is one of three aromatic amino acids that minimally contain a six-membered benzene ring in their side chains, the other two being phenylalanine (Phe, F) and tyrosine (Tyr, Y). Whereas Tyr is simply a p-hydroxy derivative of Phe, the side chain of Trp is indole, which is more complex, as it is a six-membered benzene ring fused to a five-membered pyrrole ring with an integrated NH group. Trp can also be viewed as a derivative of alanine (A), having an indole substituent on the β carbon (Table 1). The indole ring of Trp absorbs strongly in the near-ultraviolet wavelength of the spectrum, with an absorption maximum at 280 nm, which forms the basis of measuring A280 as a characteristic assay for proteins, distinguishable from nucleic acids that have an absorption maxima at 260 nm.

As presented later, the complex and nitrogenous aromatic side chain of Trp necessitates a large number of biosynthetic reactions, making Trp the most energetically expensive amino acid to synthesize. Discovered by Sir F.G. Hopkins in 1901 in milk casein hydrolysate, Trp was found to be an essential amino acid in experiments with mouse diet. Over the years, interest in Trp and its nutritional role in the mammalian diet has received enormous attention, much of which can be found in recent reviews [1,2,3,4,5,6]. Here, I have taken a complimentary approach, critically analyzing the major roles of Trp in cellular functions and in intermediary metabolism, such as translation, protein structure, adduct formation, the generation of important regulators, and most recently, RNA virus regulation. 

## 2. Trp Codon, UGG

The standard genetic codon table assigns a total of 64 trinucleotide codons to the 20 amino acids used in translation. While a majority of amino acids are coded for by a set of four synonymous codons, some (e.g., Ser, Leu, Arg) are encoded by six codons, while several (e.g., His, Tyr, Gln, Asn, Lys, Asp, Glu, Cys) are encoded by two codons. The synonymous codons are thus functionally ‘redundant’, as they code for the same amino acid. Three nonsense codons, namely UAA, UAG, and UGA, do not code for any amino acid, but rather promote translational stop, and therefore, in this function, they are redundant as well. Methionine is an exclusive amino acid, since it is the nearly universal starting residue in translation, and is encoded by a single codon, AUG, both for translation initiation and for incorporation at internal sites during elongation. It is, therefore, intriguing that the only other amino acid, and the only internal amino acid, encoded by a single nonredundant codon, is Trp, encoded by UGG.

The mechanism and implications of the redundancy of codons have been a matter of intense speculation and research since the discovery of the complete genetic code [7,8,9]. The redundancy, which generally occurs at the third position of the codon, is possible due to ‘wobble’ base-pairing, as proposed by Crick in a set of rules, known as the ‘wobble hypothesis’ [10]. The central tenet of the hypothesis is that while the first and second positions of the codon obey the classic Watson–Crick base-pairing (A:U, G:C) in codon–tRNA (codon–anticodon) recognition during translation, the third position is relatively tolerant to nucleotide mismatch. This is largely due to the structural flexibility or ‘wobble’ of the tRNA molecule [10,11,12]. It is now believed that redundancy serves as a built-in genetic safety mechanism such that errors in the third position of the codon will be relatively silent, since it will still code for the same amino acid, thus maintaining the wild-type polypeptide sequence. Even outside of the synonymous codons, single nucleotide changes in many codons may lead to conservative replacements with similar amino acids; for example, changing UCG to ACG changes Ser to Thr, hydroxy amino acids that are often functionally similar (e.g., phosphorylated/dephosphorylated by the same Ser/Thr protein kinase/phosphatase). Similarly, GAU and GAA, differing in the third position, code for the acidic amino acids, Asp and Glu, respectively, and CUU and AUU code for Leu and Ile, which are structural isomers.

The nonredundant Trp codon does not enjoy this benefit, and in fact, single nucleotide substitutions at the third position—or at any other position of Trp codon—result in amino acids of very different physiochemical and functional properties, all of which, unlike Trp, are aliphatic (Table 1). Two changes (UGA, UAG) result in stop codons. Clearly, mutations in the Trp codon are highly likely to be deleterious, which reinforces the aforesaid suggestion that Trp is used judiciously in the protein, only where it is absolutely needed for its distinctive properties, which are detailed later.

It is tempting to speculate that the rarity of the Trp codon, combined with the low intracellular concentration of Trp, will promote ribosome pausing at the Trp codons during translation, allowing the proper folding of the nascent polypeptide [13,14,15]. While this is entirely possible, it would be a premature assumption, since many other factors influence translational dynamics, such as tRNA concentration, codon usage, and RNA secondary structure, which remain to be fully studied for Trp.

## 3. Trp Biosynthesis: Salient and Unique Features

With a molecular formula of C_11_H_12_N_2_O_2_ and molecular weight of 204.22 g/mol, Trp is the largest proteinogenic amino acid in the cell. As indicated earlier, Trp is not synthesized from simple molecules in animals, including humans, and is thus essential in their diet. Essential amino acids, in general, are synthesized by plants and microorganisms, and when present in the animal diet, are derived mainly from plants. The biosynthesis of all three amino acids, viz. Phe, Tyr and Trp, initially follows a common pathway that generates chorismate, known as the shikimate pathway [16,17]. From this point, the Trp biosynthetic pathway veers away from those of Phe and Tyr, in part due to the need to construct the indole ring of Trp. Chorismate is converted into prephonate, which then bifurcates into the Phe or the Tyr synthetic pathways; in contrast, chorismate is converted to anthranilate in the Trp synthesis pathway, which is not a shared precursor for Phe or Tyr synthesis. Subsequently, four additional steps are required to produce indole. The last step of Trp biosynthesis involves the condensation of indole with serine, the smallest hydroxy amino acid, which provides the features common to all amino acids, namely the alpha-carbon and amino and carboxylic groups. This step is catalyzed by tryptophan synthase (or synthetase), a complex multisubunit enzyme that occurs in microorganisms and plants but is absent in animals. 

Trp synthase is a classic example of “substrate channeling” in which the indole is held in position within the α subunit until the Ser in the β subunit is converted to the highly reactive aminoacylate, which is followed by a fast reaction between the two [18]. Importantly, the coordinated channeling of the reactants prevents the release of indole from the enzyme core; without channeling, the indole could be released, and due to its hydrophobic nature, could traverse the cell membrane and exit out of the cell, thus abrogating Trp synthesis at the very last step.

**Figure 1:**
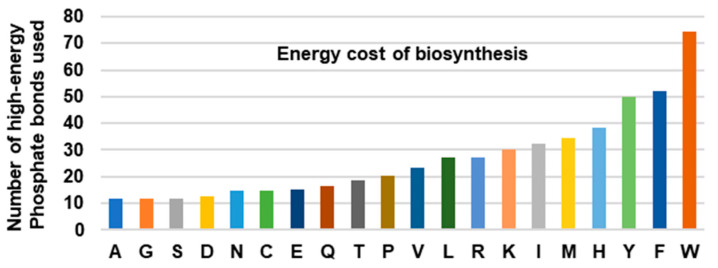
Total energy cost of amino acid biosynthesis. The total number of high-energy phosphates, equivalent to ATP and GTP, required for the biosynthesis of each amino acid, was plotted; the numbers were obtained from [19]. Note that the aromatic amino acids (Tyr, Phe, Trp) are energetically more expensive than the others, Trp being the highest of all.

The large number of reactions in the Trp biosynthetic pathway comes with a high energy bill. This was recognized early on in a pioneering study that calculated the energy cost of the twenty amino acids by adding the number of high-energy phosphate bonds (~P) required to generate the respective precursors of each pathway [19]. The bar graph, generated from the data (Figure 1), clearly reveals that the three aromatic amino acids are the most expensive to synthesize, and Trp, which requires an equivalent of 74~P bonds, tops the list by a sizeable margin over Phe (52~P) and Tyr (50~P). It has been postulated that the high energy cost is a major reason that the animals obtain the expensive amino acids from the diet instead of synthesizing them.

## 4. Metabolism of Trp

### 4.1. Trp Degradation Pathways and the Gateway Enzymes

**Figure 2:**
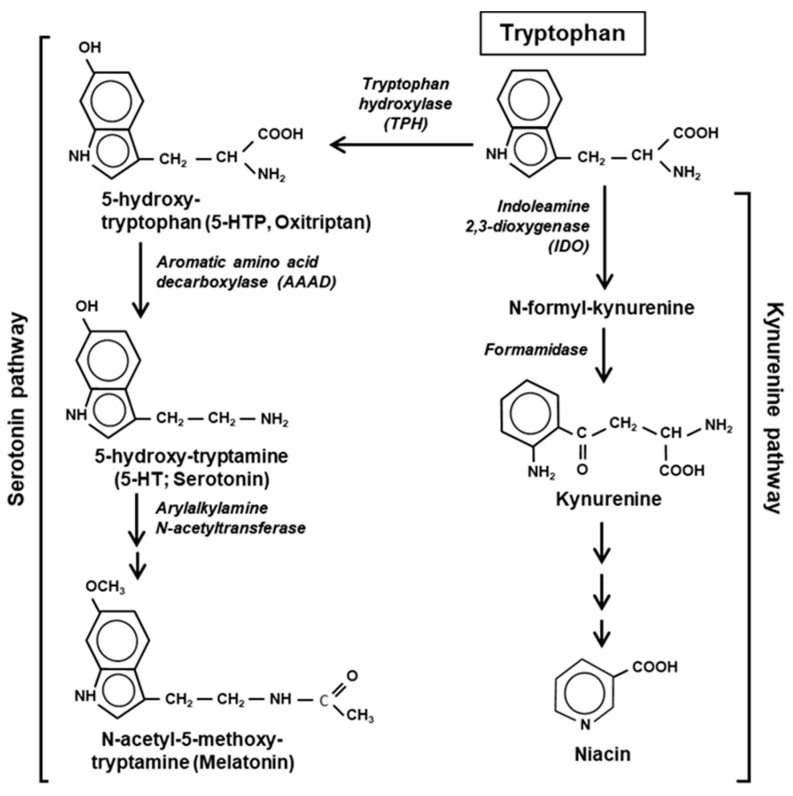
Two major pathways of Trp degradation. The serotonin and kynurenine pathways with their enzymes and products are shown. Note that the indole ring of Trp is destroyed at the very first step of the kynurenine but persists through the serotonin pathway.

The major pathways of Trp catabolism and conversion to the key secondary metabolites are summarized here in a simplified diagram (Figure 2).

Tryptophan is degraded via two parallel pathways, which can be named by their respective products or intermediates, viz. the serotonin pathway and the kynurenine pathway. These two pathways are non-overlapping and mutually exclusive, but differ in activity, the kynurenine pathway being substantially more active in mammalian cells. The two pathways compete with each other for the available pool of free Trp, not used in translation [20]. Fortunately, due to the low frequency of Trp residues in the proteins, nearly 99% of the total cellular Trp is available for non-translational use (Section 7). The serotonin pathway is initiated by tryptophan hydroxylase (TPH) that adds a hydroxy group to the 5 position of Trp to generate 5-HTP, also called oxitriptan. The kynurenine pathway is initiated by either of the two heme-containing oxidoreductases, indoleamine 2,3-dioxygenase (IDO) or the highly related tryptophan 2,3-dioxygenase (TDO). These two first steps are also the slowest—and hence rate-limiting—steps of the respective pathways, acting as gateways of Trp recruitment. An important distinction between the two pathways, which was noted earlier [21], is that the kynurenine pathway destroys the aromatic indole core of Trp, while the serotonin pathway retains the aromatic structure through all the compounds of the pathway (Figure 2).

Mammals possess two isoforms of TPH, viz. TPH1 and TPH2, which are ~70% identical in sequence, differing mainly in the regulatory domains, and are expressed in a tissue-specific manner [22,23]. While TPH1 predominates in peripheral tissues that express serotonin (a neurotransmitter; see later), such as the gastric system and skin, TPH2 is mostly expressed in neuronal cell types such as the central nervous system (CNS), specifically the brain. The difference in the regulatory domains likely allows them to have tissue-specific regulations. TPH is in fact a member of the amino acid hydroxylase superfamily that also comprises phenylalanine hydroxylase (PAH) and tyrosine hydroxylase (TH), all of which possess similar active sites and uses the same cofactors, and thus, there is substantial overlap in their substrates along with preference. TPH, for example, hydroxylates both Trp and Phe with comparable kinetics; however, it hydroxylates Tyr at a ~5000-fold slower rate [23]. The full implication and molecular mechanism of the substrate overlap may shed important light on the distribution and evolution of these enzymes, which have remained unresolved.

Indoleamine 2,3-dioxygenase (IDO or IDO1), the first enzyme of the kynurenine pathway (Figure 2), serves as an important immunoregulatory checkpoint [24,25]. A closely related isoform (43% similar), referred as IDO2 [26], likely resulted from gene duplication, but is expressed in a very limited number of tissues, mainly liver, kidney and antigen-presenting cells in small amounts, and is also enzymatically much less active on Trp (~340-fold higher Km). It appears to have an accessory role in IDO1-mediated immune regulation and in inflammation [27,28]. In most literature, and in this review, the term IDO is to be considered synonymous to IDO1.

### 4.2. Secondary Metabolites of Trp

Because of the limiting concentration of intracellular Trp, it is reasonable to assume that the Trp degradation pathways indirectly regulate Trp levels by diverting some of the Trp to secondary metabolites. Thus, as in translation, Trp is frugally used in degradation, only to generate metabolites that are physiologically important, which will be briefly discussed here. Several metabolic products of Trp have received significant attention, the notable ones being 5-HTP (oxitriptan), serotonin (a neurotransmitter), melatonin, kynurenine, niacin (vitamin B3), and tryptophol. While melatonin and niacin are end-products of the two parallel pathways described above, 5-HTP, serotonin and kynurenine are intermediate metabolites (Figure 2). The dietary and pharmacological aspects of these chemicals have been extensively researched and reviewed, and therefore, only their molecular and regulatory roles and their relationship to Trp will be summarized, with emphasis on the underlying mechanisms, where available.

#### 4.2.1. Metabolites of the Serotonin Pathway

Often branded as ‘oxitriptan’, 5-hydroxytryptophan (5-HTP) is a naturally occurring non-proteinogenic amino acid. As an immediate precursor of serotonin, a well-known monoamine neurotransmitter, 5-HTP is sold around the globe under many brand names as an over-the-counter (OTC) sleeping aid [29] as well as a suppressor of depression and appetite. Many clinical users prefer 5-HTP as it crosses the blood–brain barrier, whereas serotonin does not.

Serotonin (5-hydroxytrypatime) exhibits a multiplicity of complex physiological and clinical effects in diverse tissues, modulating mood, cognition, learning, and vasoconstriction [30,31]. Most of the regulatory roles of serotonin are triggered by binding to a large family of cell-surface receptors, known as serotonin receptors or 5-HT receptors [32,33]. However, serotonin can also cause the receptor-independent regulation of proteins through the post-translational addition of the serotonin moiety, in a process called ‘serotonylation’, in which the transglutaminase enzyme creates a glutamyl-amide bond between the primary amine group of serotonin and the carboxyl group of a glutamine residue in the acceptor protein [34,35,36]. The expanding list of serotonylated proteins regulate a variety of processes, such as thrombocyte production, vascular smooth muscle contraction, pulmonary hypertension, and release of insulin from pancreatic β cells [36].

In the human body, nearly 90% of serotonin is located in the enterochromaffin cells in the gastrointestinal tract, where it regulates intestinal movements, essential for the proper travel of food [30,32]. The 1–2% that is found in the CNS participates in the control of mood, sleep and hunger. Serotonin also regulates several cognitive functions, such as memory and learning. Drugs that alter serotonin levels of the body are used to treat a variety of psychiatric disorders, such as depression, anxiety, poor memory, migraine, nausea, and phobia, as well as sometimes obesity and Parkinson’s disease [33]. Several serotonergic psychedelic drugs—naturally occurring as well as synthetic controlled substances—are agonists of 5-HT receptors [31].

Nonetheless, the pharmacological use and benefit of both 5-HTP and 5-HT (serotonin) have been matters of controversy [30,37,38], in part because many clinical studies of either metabolite lacked proper controls and were considered inconclusive in meta-analyses. Both exhibit a plethora of adverse effects, particularly in higher doses, including cardiovascular problems, upset stomach, headache, agitation, panic attacks, fatigue, sexual dysfunction, and suicidality. Not unexpectedly, similar side effects and a lack of scientific evidence also apply to the supplemental use of tryptophan, the parent compound.

Melatonin, a natural hormone, is the end-product of the serotonin pathway, and therefore, all the upstream compounds, from Trp to serotonin, can serve as its precursor. Melatonin helps to maintain the circadian clock of the body, particularly the wake–sleep cycle, comprising of ~16 h of daytime activity and ~8 h of nightly sleep [39]. It is generally safe to use and is used to treat insomnia, jet lag and various sleep disorders, thrombocytopenia (chemo-induced), ‘winter blues’ and seasonal affective disorder (SAD), and tardive dyskinesia [40,41,42,43]. Melatonin also has some immune-regulatory and anticancer effects, but these effects need further studies and validation [44,45]. Melatonin is also produced synthetically and is freely available as an OTC dietary supplement.

#### 4.2.2. Metabolites of the Kynurenine Pathway

The kynurenine pathway (Figure 2) is responsible for processing >90% of the Trp in humans, producing kynurenine and niacin as the major metabolites. The build-up of these and other metabolites of this pathway can lead to multiple pathological conditions, such as AIDS-related dementia, multiple sclerosis, and ischemic brain injury [46,47,48,49,50], although the molecular mechanisms are not fully clear.

Niacin, synonymously called nicotinic acid, is the end-product of the pathway (Figure 2), and is a form of vitamin B3, essential in human health. Niacin occurs naturally in a variety of foods, such as meat, fish, and nuts, and is used to treat pellagra, a niacin-deficiency disease that shares some symptoms with Trp-deficiency. It is commonly seen among the poor and the malnourished in sub-Saharan Africa, Indonesia and China, as also in rural South America, where the staple food is maize, which is low in Trp and niacin. Interestingly, niacin is available in two forms, nicotinic acid (or niacin), and nicotinamide (or niacinamide), both have vitamin function, in which they act as precursors of the coenzymes nicotinamide adenine dinucleotide and nicotinamide adenine dinucleotide phosphate (NAD and NADP, respectively). However, niacin is also a medicine, prescribed for lowering cholesterol and triglyceride levels. 

#### 4.2.3. Tryptophol and Related Indole Derivatives

Several biologically important indole derivatives are also produced from separate branches of Trp metabolism [51]. A major one is tryptophol or indole-3-ethanol (indole–CH_2_–CH_2_OH), an aromatic alcohol. Its biosynthetic pathway (also known as the ‘Ehrlich pathway’ after its discoverer Felix Ehrlich) begins with the deamination of Trp to 3-indole pyruvate, followed by decarboxylation to indole acetaldehyde, and then reduction to the alcohol by alcohol dehydrogenase [52]. Tryptophol is produced mainly by plants and lower eukaryotes, such as yeast, fungus, marine sponge, and the unicellular protozoan parasite, Trypanosoma brucei, the agent of the deadly African ‘sleeping sickness’. Specifically, tryptophol is found in wine and beer, as a secondary product of ethanol fermentation by yeast; it contributes to the distinctive taste of wine, and also acts as a quorum-sensing molecule for yeast population control [53]. As a strong ‘soporific’ (sleep promoting agent), it is the causative chemical of ‘sleeping sickness’, facilitated by its ability to cross the blood–brain barrier [54]; thus, it can be considered a functional analog of serotonin and melatonin, the two neuroactive products of the serotonin pathway of Trp degradation (Section 4.2.1). The 5-hydroxy and 5-methoxy derivatives of tryptophol are also sleep inducers, as tested in mice [55]. Note that the best studied plant growth hormone, auxin, is indole acetic acid (IAA), which is also produced from 3-indole pyruvate in plants, and via several other parallel pathways in *Neurospora crassa*, a filamentous fungus [52]. A small amount of Trp is converted into indole by the action of bacterial tryptophanase enzyme in the gut. A number of organisms convert tryptophol into other derivatives, such as indole acetaldehyde, and glucoside, galactoside and mannoside conjugates of unknown function [56,57,58]. Finally, tryptophol is a starting material for many natural and synthetic bioactive compounds that have been masterly described in a recent treatise [59]. A short list of the naturally occurring compounds include goniomitine, spermacoceine, and physovenine, whereas chemical synthesis generates a seemingly endless roster of products such as a novel inhibitor of hepatitis C virus NS5B polymerase, and etodolac, which in turn is a progenitor of a class of bioactive compounds with anti-inflammatory, analgesic and anticancer properties [59].

To sum up, cellular Trp metabolism generates several chemicals of paramount physiological importance that affect myriad aspects of health and behavior; however, their pharmaceutical applications and mechanism of action need further scrutiny and in-depth analysis.

#### 4.2.4. Inhibition of Gluconeogenesis by Trp

Trp and some of its metabolites have a unique role in regulating gluconeogenesis, a pathway that generates glucose from non-carbohydrate carbon substrates, including several amino acids. Gluconeogenesis in vertebrates takes place mainly in the liver, and constitutes a major mechanism that maintains blood glucose levels in times of need, such as fasting, starvation and intense exercise, thus preventing hypoglycemia. Over half-a-century ago, it was noted that tryptophan acts as an inhibitor of gluconeogenesis [60,61,62]. Biochemical studies have since revealed that Trp inhibits phosphoenolpyruvate carboxykinase, a key enzyme of the gluconeogenesis pathway [61], but the molecular mechanism of this enzymatic inhibition has remained unknown. The metabolic rationale for the unique anti-gluconeogenesis effect of Trp has also remained a matter of speculation; however, it is logical to assume that a high concentration of a usually low cellular level of Trp would indicate adequate energy charge, making the synthesis of glucose unnecessary. Regardless of the mechanism, Trp appears to act as an important regulator of glucose and energy metabolism.

## 5. Trp Adducts in Proteins

**Figure 3:**
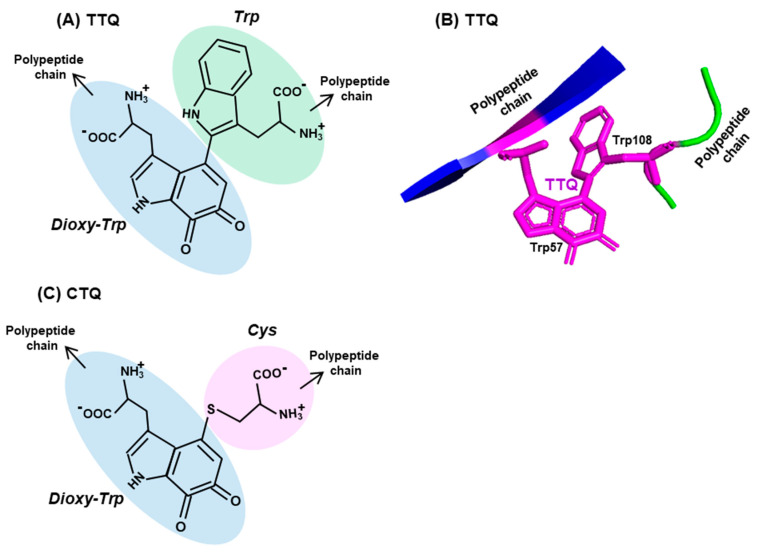
Two representative Trp adducts. Structures of two adducts, (**A**) tryptophan tryptophylquinone (TTQ) and (**C**) cysteine tryptophylquinone (CTQ) are shown, with each amino acid moieties circled in different colors (three Trp, one Cys), the dioxy-derivative of Trp being blue (compare with the Trp structure in Figure 2). Both TTQ and CTQ are part of the polypeptide chain, the continuity of which is indicated by arrowheads. PyMol representation of TTQ in the PDB entry 2BBK is shown in (**B**).

Enzyme cofactors are typically preformed organic moieties or metals that are added to the enzyme polypeptide from external sources and are reversibly and noncovalently attached. However, a class of protein-derived cofactors are formed by the post-translational modification of one or more amino acid residues [63,64,65,66,67], a prototype example of which is tryptophan tryptophylquinone (TTQ). It is a unique adduct that functions as an essential cofactor of select amine dehydrogenases, such as methylamine dehydrogenase (MADH) and aromatic amine dehydrogenase (AADH) [64,65,66]. TTQ, generated by irreversible posttranslational conjugation of two Trp residues (hence, sometimes denoted as Trp–Trp) within the same polypeptide (Figure 3A,B), forms the catalytic or redox-active center of the dehydrogenase. This role of Trp is critical to prokaryotic energy metabolism, as the MADH holoenzyme catalyzes the oxidative deamination of methylamine, eventually allowing the organism to use methylamine as the sole source of carbon, nitrogen and energy [67].

The biosynthesis of TTQ involves the cross-linking of the indole rings of two Trp residues, brought to proximity by polypeptide folding, and the insertion of two oxygen atoms onto adjacent carbons of one of the indole rings [63,64,65,66] (Figure 3A,B). The synthesis is orchestrated through a complex series of steps, within a precursor protein of MADH (called pre-MADH), and is completed by the diheme enzyme, MauG. The details of the electron transfer that occurs during these steps are beyond the scope of this review, but it is sufficient to mention that strategically located Trp residues of MauG are also active participants in this process.

Lastly, another protein-derived cofactor, cysteine tryptophylquinone (CTQ), exemplifies the similarity as well as diversity in Trp-derived cofactors [68]. CTQ resembles TTQ in structure, but the oxygenated Trp residue is crosslinked to the sulfhydryl group of a Cys residue (Figure 3C). In contrast to TTQ, which are found exclusively in dehydrogenases, CTQ is found in both dehydrogenases and oxidases [64,68,69]. As observed earlier [64,70], these are the first amino acid oxidases that do not use flavin as cofactor. Collectively, these tryptophylquinone cofactors showcase the novelty and versatility of the use of Trp in biology.

## 6. Interacting Partners of Trp Residues in A Polypeptide

### 6.1. Nature of Trp Side Chain Interactions

As briefly stated before, the uniqueness of the molecular properties of Trp among the 20 amino acids is due to a combination of the following structural features [71]: the largest nonpolar (hydrophobic) area of the two-sided π-electron face that is also polarizable and highly accessible because of its planar topology; the strongest electrostatic potential for cation–π interactions; and an indole N–H moiety that can donate a H-bond. Indeed, redox-active Trp side chains play cardinal roles in electron transfer and protein function through regulated protonation state H-bonding. Lastly, Trp exhibits distinctive and sharp Raman spectral lines that have facilitated our understanding of the molecular mechanisms of protein structure and function [72,73].

**Figure 4:**
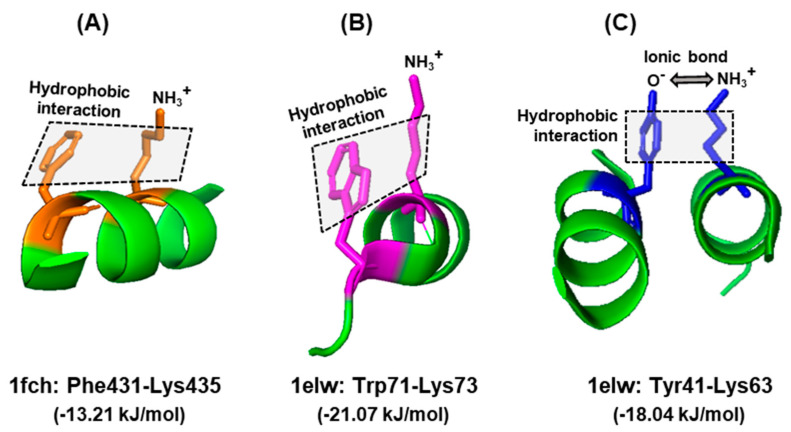
Interactions of aromatic side chains. Example of similarity and distinction among the three aromatic amino acids. In all three examples, the hydrophobic aromatic parts of the side chains of Phe (**A**), Trp (**B**), and Tyr (**C**) are engaged in hydrophobic interaction with the hydrocarbon part of Lys. In case of Tyr, the acidic (oxyanion, O^−^) terminus additionally forms an ionic bond with the amino terminus of Lys side chain (NH_3_^+^), since they are negatively and positively charged, respectively, at physiological pH. The hydrophobic interaction is denoted by shaded rectangles. Other, interconnecting interactions are not shown for clarity; for example, the positively charged amino group of Lys435, which appears to be free in this diagram, is actually engaged in ionic interactions with the negatively charged carboxylic acid group of Glu232 (not shown), brought to proximity due to folding of the polypeptide. The amino acids in each interacting pair are indicated with the same color. The structures were obtained from the PDB entries written below, along with the corresponding energy of stabilizing interaction, as described in Section 6.1. Only the relevant portions of the proteins are shown as a ribbon diagram in the PyMol presentation.

Nevertheless, there has been no large-scale studies interrogating these properties of Trp for their interaction with amino acid residues in actual protein structures. To fill this vacuum, a number of X-ray crystallographic structures of diverse proteins were retrieved from the PDB protein databank (https://www.rcsb.org), and the side chain interactions of Trp were compared with those of two other aromatic residues, namely Phe and Tyr. These residues served as ‘controls’ of one another, since all three have π–electron surfaces, but Trp is the largest, and Tyr is the only one among the three that has a polar hydroxyl group. A set of example interactions is illustrated here (Figure 4), in which the nature of bonds was viewed in the secondary structure presentation in PyMol. For brevity, a single amino acid, namely Lys, was chosen as the interacting partner of all three, and the spacing was observed from multiple angles for an optimal view. The rational for choosing Lys was that it is amphipathic, as its side chain offers both a hydrophobic stretch and a terminal polar (amino) group. Thus, it is capable of interacting with the hydrophobic section of all three amino acids and also with the hydroxy group of Tyr. The results (Figure 4) show that this is indeed the case, i.e., the predicted hydrophobic and the ionic interactions could be discerned in appropriate spatial conformation.

Note that this is only a portion of the interactions, relevant for this query, and that each residue may interact with multiple other entities, such as water molecules, ions, and other neighboring residues. For example, the nitrogen (NH) of the indole ring can donate a hydrogen bond, and as a result, Trp can also facilitate the solvation of folded proteins, which cannot occur with Phe. Thus, the Trp side chain has a dipole moment of ~2D [74], whereas the Phe side chain does not have any. Lastly, cation–π interactions between the indole ring and Lys/Arg also stabilize a structure, provided they are in the appropriate spatial location. Nonetheless, the contribution of various environmental and structural parameters in a larger sample set is required to explore the significance of the energy difference patterns (also see Section 6.2).

### 6.2. The Energy Landscape of Trp Side Chain Interactions

**Figure 5:**
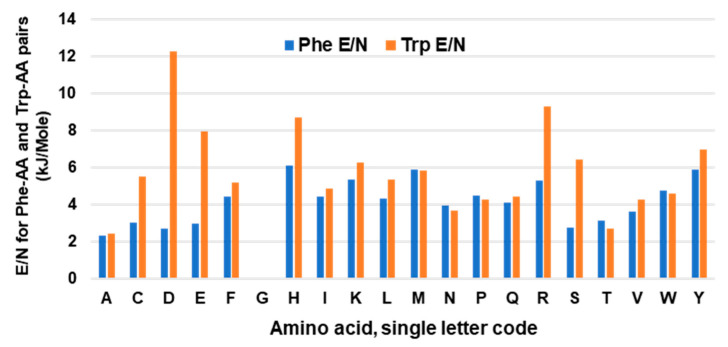
Average energy per interacting pair of amino acids. Energy of interaction of Trp or Phe with other amino acids were collected from multiple proteins, and the total energy (E) of a pair was divided by the total number of the pair (N) and plotted, as described in Section 6.2. Note the absence of values for Phe–Gly and Trp–Gly as Gly lacks a side chain.

The stabilizing free energy of interaction between amino acid side chains promotes and maintains the optimal higher order structure of a polypeptide backbone. These studies were, therefore, extended to analyses of the free energy operating between side chains involving Trp in several randomly selected proteins. The energy values were collected from the ‘interaction energy matrix web server’, described previously [75,76], manually entered in Excel, and those for Phe and Trp were separated out for comparison, since both are aromatic and hydrophobic. For proof-of-concept analysis, values for a total of 202 Phe and 92 Trp residues were tabulated. To illustrate, the 202 Phe residues interacted with a total of 105 Ala residues (N), and the total energy of the stabilizing interactions (E) was 244.16 kiloJoules/mole (kJ/mol). The energy per residue was then calculated as E/N, and this was similarly calculated for each of the 20 amino acids. In this manner, the strength of interaction between a pair of residues could be compared regardless of the total number of such pairs. The plotted results (Figure 5) revealed a similar interaction ensemble for Phe and Trp, i.e., their partner preferences were similar. Trp showed a higher E/N value for several interacting residues, but most notably for D and E. Interestingly, these residues, although acidic overall, possess hydrocarbon portions, which may promote stronger hydrophobic interactions with the larger hydrophobic area of Trp, as shown for Lys–Trp and Lys–Phe.

**Figure 6:**
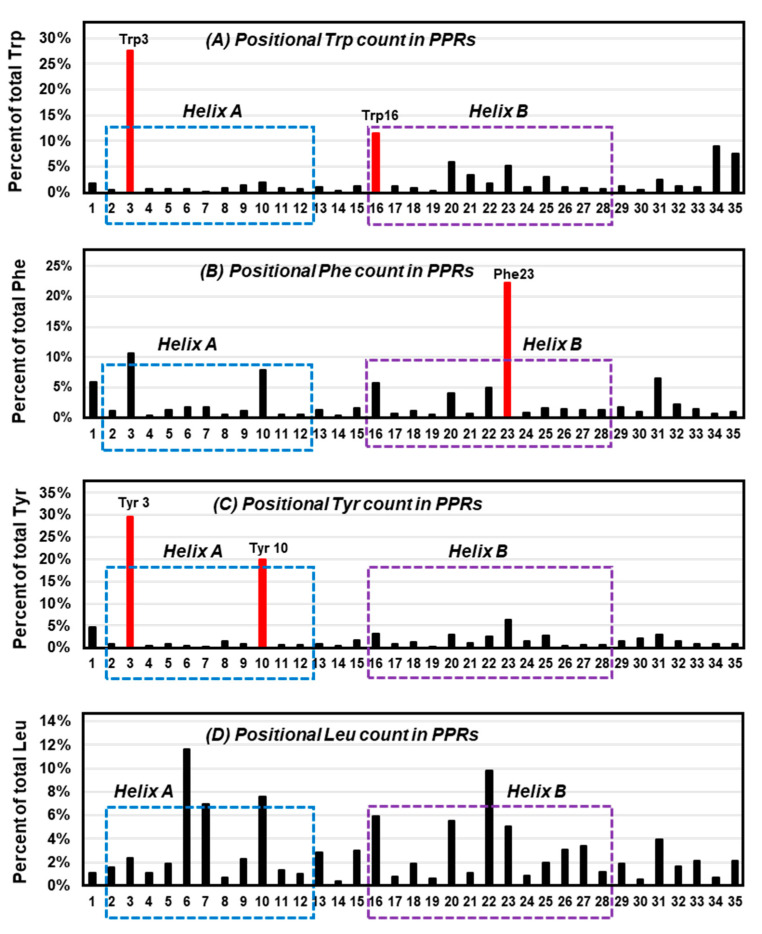
Locations of selected amino acids in all-helical repeats. The previously published collection of bihelical pentatricopeptide repeat (PPR) sequences [77] were visually analyzed for the location (1 to 35) of three aromatic residues, namely Trp (**A**), Phe (**B**), Tyr (**C**), and the control, nonaromatic but hydrophobic residue, Leu (**D**), and their percentage of occurrence in each position of the repeat was plotted. The most abundant location(s) of the aromatic amino acids are shown in red (Trp3/16, Phe23, Tyr3/10) and are indicated as red bars; note that Tyr3 and Trp3/16 tend to reside near the N-terminal end of the helices. Helix A and helix B [77,80] are indicated by dotted boxes, respectively, blue and purple.

Since the overall interaction energy survey did not show a clearly distinctive profile for Trp, it was inquired if perhaps the location of Trp in proteins exhibits a conspicuous signature. To this end, a previously collected large set of 22,999 pentatricopeptide (35 amino acid) repeats (PPRs) [77] was analyzed. The PPR is a class of nonidentical 35-amino acid-long repeats that contain signature amino acids in specific positions, and consist of repeat bihelical structures connected by flexible loops [77,78,79]. In previous studies, the Trp residues in the PPR were shown to be concentrated at residue numbers 3 and 16 [77]. Here, a similar study was performed for Phe and Tyr, and Leu as a dissimilar ‘control’, on the same set of PPRs, and the results were plotted for each (Figure 6), which revealed amino acid-specific patterns.

Trp was confirmed to occur at positions 3 and 16 (Figure 6A), which are near the beginning of the two signature PPR helices, designated as helix A and helix B [77]. In contrast, Phe was mainly concentrated at 23 (Figure 6B), and Tyr at 3 and 10 (Figure 6C). In other words, position 3 was popular with both Trp and Tyr, but not so much with Phe. Although the exact significance of the placement remains to be determined, in the more extensively studied and highly related tetratricopeptide repeats (TPRs) [80] the analogous Trp residue was shown to make extensive packing interactions with Leu in the preceding helix and H-bonding (by the ring NH) to the backbone carboxylate of the signature Pro32 in the following helix (not shown in Figure 2). A nonaromatic amino acid, Leu, showed no preference for any of those ‘aromatic’ positions, but was distributed over several other places (Figure 6D). It thus appears that Trp has its own preference for specific positions in these bihelical repeats, distinct from the others.

### 6.3. Trp in Membrane Proteins and Antimicrobial Peptides

As indicated earlier, even though Trp is hydrophobic overall, the indole NH moiety can donate a hydrogen bond, and allow it to interact with the aqueous solvent. Moreover, cation–π interactions, such as when Lys or Arg is close to an aromatic ring, can provide the energy stabilization of several kilocalories/mole, which is often stronger than ionic bridges [81,82]. The combination of the physical properties of Trp makes it an ideal amphiphilic residue for the hydrophobic/hydrophilic interface of membrane proteins, where it likely plays functionally important roles [83]. In fact, while Trp is the least abundant residue in soluble proteins, accounting for only 1.1% of the amino acids expressed in cytoplasmic proteins, it is more prevalent in membrane proteins, with an abundance of 2.9% in transmembrane α-helical domains [84,85]. Several studies, including the pioneering use of UV Raman resonance spectroscopy, revealed that Trp residues act as anchors along the lipid bilayer interface, which stabilizes membrane-spanning proteins [86,87,88].

**Figure 7:**
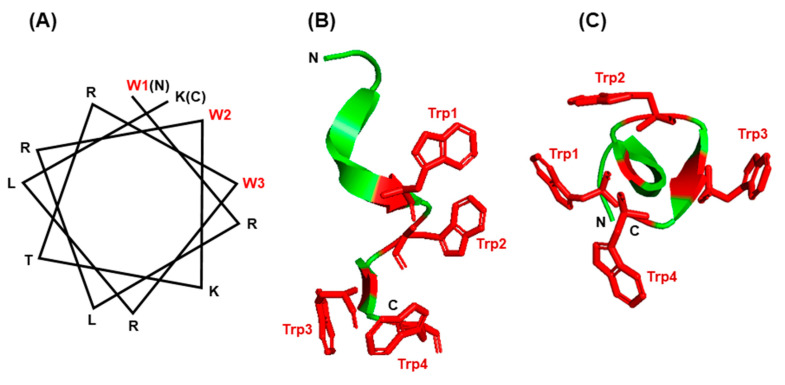
Structure of small Trp-containing peptides. (**A**) Helical wheel presentation of peptide WRK-12 (WRLRWKTRWRLK) and (**B**,**C**) three-dimensional structures of gramicidin A (PBD 1C4D) in PyMol ribbon presentation are shown. All Trp (W) residues are indicated in red. For gramicidin, two views of the Trp-containing portion of the peptide are presented: a side view (**B**) and a top view of the cylinder shape (**C**), showing the Trp side chains protruded around the channel. The amino- and carboxy-termini are indicated with N and C, respectively.

An impressive body of literature has documented that indole forms hydrogen bonds in membrane-associated proteins and peptides [88,89,90,91,92,93]. In the bacterial β-barrel outer membrane protein A (OmpA), mutating the Trp residues to Phe in fact destabilized the protein when folded into lipid bilayers [87,94]. The unique properties of Trp have been harnessed by a large family of short peptides with broad-spectrum antimicrobial activity, commonly referred to as ‘antimicrobial peptides’ or AMPs, essential for host defense and survival. Many AMPs also possess multiple other biological functions, and are therefore, also called host defense peptides (HDPs), such as the ability to regulate inflammation and immunity [93,95]. In several of them, such as members of the ‘temporin’ and ‘aurein’ family, the pairing of Trp and Arg has been shown to be essential for superior activity and bioavailability, as reviewed recently [95]. The Trp/Arg amino acid pair is a common theme in many AMPs, where it allows for cation–π interaction as well as the unique side chain properties of the Trp indole ring, mentioned earlier, thereby promoting the formation of the proper higher order structure and interaction with membrane lipid bilayer. A large number of diverse temporins and aureins, secreted from the skin of several frog species, protect these amphibians from infections in the wild. In another example, short Trp-rich AMPs provide antimicrobial defense to the water buffalo (*Bubalus bubalis*) [96] and have been subjected to mutational analysis. An optimally designed variant, WRK-12 (WRLRWKTRWRLK), was shown to efficiently target LPS and bacteria-specific phospholipids on the membrane [97], and as with many other AMPs, the placement of the Trp residues on one face of the helix (Figure 7A) was crucial for amphiphilicity and membrane interaction.

The channel peptide antibiotic, gramicidin A, has served as a prototype peptide for protein–lipid interactions [98]. Gramicidin A is an amphiphilic AMP consisting of 15 amino acids, of which four are Trp. The gramicidin family has several members of differing sequence, but common structural and functional features. All gramicidins exhibit a complex ensemble of structures, but in all of them the Trp residues are clustered on the surface of a helical conformation (Figure 7B,C), so that gramicidin locates at the membrane interface in the channel. As the Trp residues are substituted by Phe the channel conductance of the substituted gramicidin decreases along with a loss of antibacterial activity [99,100]. In other words, all four Trp residues are required for the full functionality of gramicidin A [101]. The formation of H-bond by indole NH, which cannot occur with Phe, is also essential for the native conformation and function of gramicidin and other ion channels and membrane proteins [102]. Ab initio calculations have also shown that the strength of the H-bonds formed between a proton donor and the π electron cloud of aromatic side chains, which represent a major class of stabilizing interactions, follow the order Trp > His > Tyr~Phe, i.e., Trp forms the strongest such bond [103]. Collectively, the examples presented in this section illustrate that the side chain features, location, and environment of Trp residues are all important for the folding and insertion of membrane proteins and membrane-associated peptides.

### 6.4. The Trp-Cage Family of Fast-Folding Peptides

Through studies of specific fragments of a naturally occurring 39-amino acid peptide, isolated from the oral secretions of a lizard species, Neidigh et al. designed a 20-residue peptide that efficiently achieved a compact and stable tertiary fold [104,105]. The folded structure was eventually named ‘Trp cage’ as its hydrophobic core shielded the side chain of the single Trp residue from solvent exposure. Kinetic measurements revealed that complete folding occurred within ~4 μs [106], establishing the Trp cage peptide as the fastest folding peptide known.

**Figure 8:**
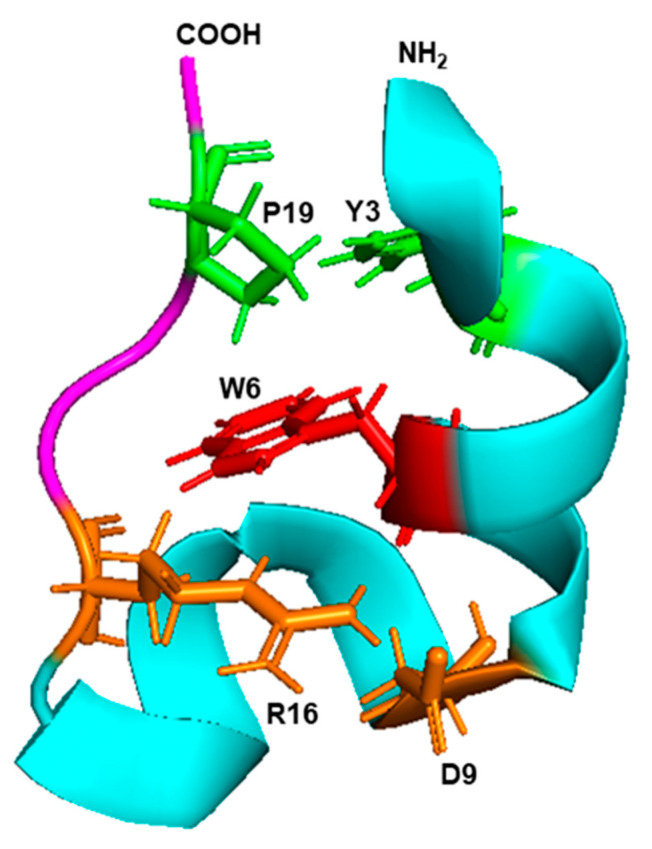
Trp cage fold. The 3D structure (PDB: 2JOF) of the 20 amino acid-long Trp cage peptide of the sequence DAYAQWLKDGGPSSGRPPPS was retrieved from the NCBI structure bank and shown in PyMol presentation. The amino- and carboxy-terminal ends are marked as such, and the single α-helix and the β-strand are colored in cyan and magenta, respectively. The central Trp (W6) is red, the Y3–P9 interacting pair is green, and the D9–R16 interacting pair is orange, as detailed in the text (Section 6.3).

Although the detailed mechanism of the Trp-cage folding is still being researched, biophysical and molecular dynamics simulation studies implicated the side chain rotamer state of the Trp residue as a major contributor to the unusually fast folding rate. Subsequently, various residues of the Trp cage were mutated and several variations of the original Trp cage sequences were also synthesized, as summarized before [107]. Collectively, this led to the consensus (Figure 8) that the Trp indole ring forms the center hub of the cage, and the rest of the peptide, which consists of just one α-helix and one β-strand on either side of the Trp, forms a hydrophobic interior that cradles this side chain to shield it from the solvent. Several helix–strand interactions hold the cage together, but the notable ones are the hydrophobic interaction between Tyr3 and Pro19, the salt bridge between Asp9 and Arg16, and several H-bonded side chains, notably the one contributed by the -OH group of Ser14 (Figure 8). The hydrophobic large indole ring of Trp was crucial for the cage structure, as the peptides in which Trp6 was replaced by His or Phe, remain largely unfolded [108].

## 7. Intersection between Trp Degradation and Immunity

Perhaps the most enigmatic aspect of Trp metabolism that has defied a clear molecular explanation for years is its intersection with various normal and pathological conditions, such as cancer, multiple sclerosis, transplantation, peripheral and CNS disorders, and recently in SARS-CoV-2 infection [47,48,49,50,109,110,111]. For the sake of brevity, the focus in this review is on selected branches of immune regulation, a key character in which is indole 2,3-dioxygenase (IDO), the rate-limiting first enzyme in the kynurenine pathway of Trp degradation (Figure 2).

It has been noted that only about 1% of dietary Trp is actually used in translation, in part due to its rarity in the polypeptides (~1.2% of total amino acids in the proteome) [9,84], while the rest is degraded or converted to multiple other compounds. As alluded to earlier, this makes Trp one of the largest contributors of non-amino acid metabolites in the cell. Approximately 90% of dietary Trp is metabolized through the kynurenine pathway alone (Figure 2), and the serotonin pathway utilizes another 1–2% [112].

IDO is strongly induced in inflammation; in fact, one of the best inducers of IDO is interferon-γ (IFN-γ) [21], which led to its designation as an “interferon-stimulated gene” (ISG). It was initially thought that IDO was a regulator of inflammatory response, and this was due to the depletion of Trp; however, the addition of Trp reversed the effect in some cell lines only, but not in others, suggesting that the mechanism is more complex [113,114]. Subsequent research demonstrated that IDO1-expressing immune cells, such as macrophages and dendritic cells, inhibit T-cell proliferation [115,116,117]. Mechanistic studies indicated that the T cell inhibition is caused by Trp depletion-induced GCN2 and/or mTOR signaling pathways. Studies in cell culture also demonstrated that exogenously added kynurenine acts as an immunosuppressive metabolite in combination with transforming growth factor. It thus appears that IDO, kynurenine accumulation, and Trp depletion, together with metabolic regulation, work together for the observed immunosuppressive effect of IDO [50,118], which is still an area of fervent research. Readers interested in further details may read the original papers, starting with the references cited here. 

In its antimicrobial role, IDO was originally found to inhibit the replication of the protozoan parasite Toxoplasma gondii in immune cells [119,120,121]. Subsequently, an antiviral role of IDO was demonstrated against measles, herpes simplex, hepatitis B, influenza and respiratory syncytial viruses [122,123,124,125,126,127]. These studies were performed mostly in cells of immune origin, such as macrophages and dendritic cells, and thus an immune-regulatory role of IDO could be at play. 

Very recently, a novel role antiviral of IDO was reported in cultured A549 cells, which are nonimmune, lung epithelial cells [21]. The induction of IDO by treatment with IFN-γ or by the expression of recombinant IDO in these cells was found to strongly inhibit the growth of human parainfluenza virus (PIV3), a negative-strand RNA virus and a major cause of sickness and death in children, the elderly, and the immunocompromised. The authors used several approaches to demonstrate that 5-hydoxytryptophan (5-HTP), the first product of the serotonin pathway of Trp degradation and the immediate precursor of serotonin, is essential to protect PIV3 growth against IDO in cell culture. The apparent antiviral effect of IDO on PIV3 growth, therefore, was not due to the generation of any of the kynurenine pathway metabolites, but rather due to the depletion of intracellular Trp by IDO, as a result of which Trp became unavailable for the alternative, serotonin pathway [21]. These studies established 5-HTP as a proviral Trp metabolite for PIV3 that is cell-intrinsic and not dependent on active immunity or humoral response. The mechanism of this proviral role of 5-HTP and its generality for other viruses remain to be determined.

## 8. Summary and Conclusions

**Figure 9:**
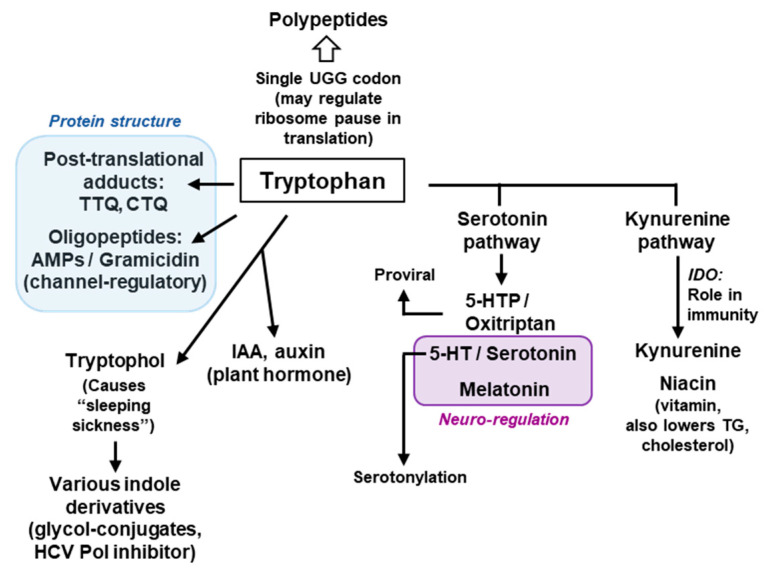
Brief schematic summary of the major roles of Trp. This is a concise diagram, showing only a few selected contributions of Trp, its metabolic products and their major roles and common usage. All compounds and their abbreviated names have been described at appropriate places in the review. The two-colored boxes indicate the main functions of the boxed Trp products, namely, the role in protein structure (Blue) and neuro-regulation (Brown). HCV = Hepatitis C virus. Note that this is by no means an exhaustive list and that many compounds have multiple roles, and many roles are played by multiple compounds, which are not shown here for the sake of brevity. AMP = Antimicrobial peptide.

This review offers a glimpse of the manifold uniqueness of tryptophan in biology, the major areas of which are summarized in Figure 9. The uniqueness of Trp in a polypeptide sequence derives from the side chain indole ring with its binuclear aromatic structure that allows it to find large hydrophobic pockets and support interactions that require a relatively large surface area. This is particularly evident in the preponderance of Trp in membrane proteins, membrane-active toxins and channels (Section 6.3), and in the formation of Trp cage (Section 6.4) and Trp adducts such as TTQ and CTQ (Section 5). It is not always clear if the indole ring also plays a pivotal role in the nearly two dozen physiologically functional small molecule metabolites that are derived from Trp, but it likely is, judging by the fact that many of them lose their bioactivity when the ring structure is opened up. For example, all three products of the serotonin pathway of Trp degradation (oxitriptan, serotonin, melatonin) (Figure 9) possess neuroregulatory and psychedelic activities, but all the products of the parallel kynurenine pathway retain only the six-membered benzene ring of indole and lack these activities.

The essential role of 5-HTP for the optimal replication of PIV3 was an unpredictable discovery that escaped attention for years, in part because the main focus was on the immunoregulatory role of kynurenine in the parallel pathway, operative in cells of myeloid origin [128]. The PIV3 studies are highly relevant for a potential antiviral regimen since PIV3 and many other negative-strand RNA viruses infect and grow in the nonimmune cells as their primary target, such as airway epithelial cells [129,130]. If 5-HTP is found to regulate the positive-strand RNA viruses as well, this could be pursued as a potential antiviral strategy against many newly emergent viruses that are lethal, such as hepatitis C, West Nile, dengue, and SARS-CoV. Lastly, the use of specific enzyme inhibitors of the two degradation pathways [21] strongly suggested that the steady-state level of 5-HTP is regulated by several factors; it is elevated by dietary Trp and TPH enzyme activity, and reduced by IDO and aromatic amino acid decarboxylase (AAAD) enzyme activity (Figure 2). The quantification of these balancing activities and their effects on virus replication under physiological and pathological conditions can be rewarding areas of future research of fundamental and clinical importance.

Since Trp is bioenergetically the most expensive amino acid, it makes sense that it is incorporated in exclusive sites in the protein where it is absolutely needed for protein structure and function, which is consistent with its rarity in proteins and its single codon. In parallel to its rare use in translation, Trp is used to generate a plethora of secondary metabolites. In other words, the translational and non-translational uses of Trp are mutually exclusive and likely compete with each other, since Trp is also the least abundant amino acid in the cell. It is tempting to wonder if Trp has other physiological roles that are still awaiting discovery.

## Figures and Tables

**Table 1:** Codons that differ from the Trp codon in the third (wobble) base.

Codon	Amino Acid	Structure of Side Chain (-R)	Distinguishing Property
UGG	Trp	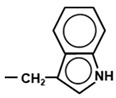	Aromatic, hydrophobic
GGG	Gly	None	Smallest, flexible, no side chain
UCG	Ser	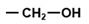	Hydroxyl, hydrophilic
UGC, UGU	Cys	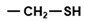	Thiol, hydrophilic (Ser homolog)
UUG	Leu	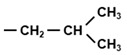	Large, hydrophobic
AGG, CGG	Arg	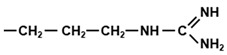	Basic, charged, hydrophilic

## Abbreviations

AAAD	Aromatic amino acid decarboxylase
AMP	Antimicrobial peptide
CNS	Central nervous system
CTQ	Cysteine tryptophylquinone
5-HT	5-hydroxy-tryptamine
5-HTP	5-hydroxy-tryptophan
IDO	Indole 2,3-dioxygenase
IFN	Interferon
LPS	Lipopolysaccharide
MADH	Methylamine dehydrogenase
OTC	Over the counter (non-prescription)
PIV3	Parainfluenza virus Type 3
PPR	Pentatricopeptide repeat
TDO	Tryptophan 2,3-dehydrogenase
TH	Tyrosine hydroxylase
TPH	Tryptophan hydroxylase
TPR	Tetratricopeptide
TTQ	Tryptophan tryptophylquinone

## References

[1] Wu G., Bazer F.W., Dai Z., Li D., Wang J., Wu Z. (2014). Amino acid nutrition in animals: Protein synthesis and beyond. Annu. Rev. Anim. Biosci..

[2] Binks H., Vincent G.E., Gupta C., Irwin C., Khalesi S. (2020). Effects of diet on sleep: A narrative. Nutrients.

[3] Chojnacki C., Popławski T., Chojnacki J., Fila M., Konrad P., Blasiak J. (2020). Tryptophan intake and metabolism in older adults with mood disorders. Nutrients.

[4] Kikuchi A.M., Tanabe A., Iwahori Y. (2020). A systematic review of the effect of L-tryptophan supplementation on mood and emotional functioning. J. Diet. Suppl..

[5] Simonson M., Boirie Y., Guillet C. (2020). Protein, amino acids and obesity treatment. Rev. Endocr. Metab. Disord..

[6] Taleb S. (2019). Tryptophan dietary impacts gut barrier and metabolic diseases. Front. Immunol..

[7] Khorana H.G., Buchi H., Ghosh H., Gupta N., Jacob T.M., Kossel H., Morgan R., Narang S.A., Ohtsuka E., Wells R.D. (1966). Polynucleotide synthesis and the genetic code. Cold Spring Harb. Symp. Quant. Biol..

[8] Nirenberg M., Caskey T., Marshall R., Brimacombe R., Kellogg D., Doctor B., Hatfield D., Levin J., Rottman F., Pestka S. (1966). The RNA code and protein synthesis. Cold Spring Harb. Symp. Quant. Biol..

[9] Gardini S., Cheli S., Baroni S., Di Lascio G., Mangiavacchi G., Micheletti N., Monaco C.L., Savini L., Alocci D., Mangani S. (2016). On Nature’s strategy for assigning genetic code multiplicity. PLoS ONE.

[10] Crick F.H.C. (1966). Codon-anticodon pairing: The wobble hypothesis. J. Mol. Biol..

[11] Limmer S., Reif B., Ott G., Arnold L., Sprinzl M. (1996). NMR evidence for helix geometry modifications by a G-U wobble base pair in the acceptor arm of *E. coli* tRNA(Ala). FEBS Lett..

[12] Murphy I.V., Frank V., Ramakrishnan V. (2004). Structure of a purine-purine wobble base pair in the decoding center of the ribosome. Nat. Struct. Mol. Biol..

[13] Ikeuchi K., Izawa T., Inada T. (2018). Recent progress on the molecular mechanism of quality controls induced by ribosome stalling. Front. Genet..

[14] Bitran A., Jacobs W.M., Zhai X., Shakhnovich E. (2020). Cotranslational folding allows misfolding-prone proteins to circumvent deep kinetic traps. Proc. Natl. Acad. Sci. USA.

[15] Collart M.A., Weiss B. (2020). Ribosome pausing, a dangerous necessity for co-translational events. Nucleic Acids Res..

[16] Braus G.H. (1991). Aromatic amino acid biosynthesis in the yeast *Saccharomyces cerevisiae*: A model system for the regulation of a eukaryotic biosynthetic pathway. Microbiol. Rev..

[17] Radwanski E.R., Last R.L. (1995). Tryptophan biosynthesis and metabolism: Biochemical and molecular genetics. Plant Cell.

[18] Miles E.W. (2013). The tryptophan synthase α2β2 complex: A model for substrate channeling, allosteric communication, and pyridoxal phosphate catalysis. J. Biol. Chem..

[19] Akashi H., Gojobori T. (2002). Metabolic efficiency and amino acid composition in the proteomes of *Escherichia coli* and *Bacillus subtilis*. Proc. Natl. Acad. Sci. USA.

[20] Le Floc’h N., Otten W., Merlot E. (2011). Tryptophan metabolism, from nutrition to potential therapeutic applications. Amino Acids.

[21] Rabbani M.A.G., Barik S. (2017). 5-Hydroxytryptophan, a major product of tryptophan degradation, is essential for optimal replication of human parainfluenza virus. Virology.

[22] Walther D.J., Peter J.-U., Winter S., Höltje M., Paulmann N., Grohmann M., Vowinckel J., Alamo-Bethencourt V., Wilhelm C.S., Ahnert-Hilger G. (2003). Serotonylation of small GTPases is a signal transduction pathway that triggers platelet α-granule release. Cell.

[23] Roberts K.M., Fitzpatrick P.F. (2013). Mechanisms of tryptophan and tyrosine hydroxylase. IUBMB Life.

[24] Yoshida R., Urade U., Tokuda M., Hayaishi O. (1979). Induction of indoleamine 2,3-dioxygenase in mouse lung during virus infection. Proc. Natl. Acad. Sci. USA.

[25] Munn D.H., Mellor A.L. (2013). Indoleamine 2,3 dioxygenase and metabolic control of immune responses. Trends Immunol..

[26] Ball H.J., Sanchez-Perez A., Weiser S., Austin C.J.D., Astelbauer F., Miu J., McQuillan J.A., Stocker R., Jermiin L.S., Hunt N.H. (2007). Characterization of an indoleamine 2,3-dioxygenase-like protein found in humans and mice. Gene.

[27] Metz R., Smith C., DuHadaway J.B., Chandler P., Baban B., Merlo L.M.F., Pigott E., Keough M.P., Rust S., Mellor A.L. (2014). IDO2 is critical for IDO1-mediated T-cell regulation and exerts a non-redundant function in inflammation. Int. Immunol..

[28] Merlo L.M.F., DuHadaway J.B., Montgomery J.D., Peng W.-D., Murray P.J., Prendergast G.C., Caton A.J., Muller A.J., Mandik-Nayak M. (2020). Differential roles of IDO1 and IDO2 in T and B cell inflammatory immune responses. Front. Immunol..

[29] Monti J.M. (2011). Serotonin control of sleep-wake behavior. Sleep. Med. Rev..

[30] Berger M., Gray J.A., Roth B.L. (2009). The expanded biology of serotonin. Ann. Rev. Med..

[31] Nichols D.E. (2016). Psychedelics. Pharmacol. Rev..

[32] Gershon M.D. (2004). Review article: Serotonin receptors and transporters–oles in normal and abnormal gastrointestinal motility. Aliment. Pharmacol. Ther..

[33] Hoyer D. (2020). Targeting the 5-HT system: Potential side effects. Neuropharmacology.

[34] Sarkar N.K., Clarke D.D., Waelsch H. (1957). An enzymically catalyzed incorporation of amines into proteins. Biochim. Biophys. Acta.

[35] Walther D.J., Peter J.U., Bashammakh S., Hörtnagl H., Voits M., Fink H., Bader M. (2003). Synthesis of serotonin by a second tryptophan hydroxylase isoform. Science.

[36] Bader M. (2019). Serotonylation: Serotonin signaling and epigenetics. Front. Mol. Neurosci..

[37] Javelle F., Lampit A., Bloch W., Häussermann P., Johnson S.L., Zimmer P. (2020). Effects of 5-hydroxytryptophan on distinct types of depression: A systematic review and meta-analysis. Nutr. Rev..

[38] Shaw K.A., Turner J., Del Mar C. (2002). Tryptophan and 5-Hydroxytryptophan for depression. Cochrane Database Syst. Rev..

[39] Claustrat B., Leston J. (2015). Melatonin: Physiological effects in humans. Neurochirurgie.

[40] Rios E.R.V., Venâncio E.T., Rocha N.F.M., Woods D.J., Vasconcelos S., Macedo D., de Sousa F.C.F., Fonteles M.M.F. (2010). Melatonin: Pharmacological aspects and clinical trends. Int. J. Neurosci..

[41] Baglioni C., Bostanova Z., Bacaro V., Benz F., Hertenstein E., Spiegelhalder K., Rücker G., Frase L., Riemann D., Feige B. (2020). A systematic review and network meta-analysis of randomized controlled trials evaluating the evidence base of melatonin, light exposure, exercise, and complementary and alternative medicine for patients with insomnia disorder. J. Clin. Med..

[42] Srinivasan V., Spence D.W., Pandi-Perumal S.R., Trakht I., Cardinali D.P. (2008). Jet lag: Therapeutic use of melatonin and possible application of melatonin analogs. Travel Med. Infect. Dis..

[43] Pandi-Perumal S.R., Srinivasan V., Maestroni G.J., Cardinali D.P., Poeggeler B., Hardeland R. (2006). Melatonin: Nature’s most versatile biological signal?. FEBS J..

[44] Kong X., Gao R., Wang Z., Wang X., Fang Y., Gao J., Reiter R.J., Wang J. (2020). Melatonin: A potential therapeutic option for breast cancer. Trends Endocrinol. Metab..

[45] Bondy S.C., Campbell A. (2020). Melatonin and regulation of immune function: Impact on numerous diseases. Curr. Aging Sci..

[46] Schwarcz R., Bruno J.P., Muchowski P.J., Wu H.Q. (2012). Kynurenines in the mammalian brain: When physiology meets pathology. Nat. Rev. Neurosci..

[47] Proietti E., Rossini S., Grohmann U., Mondanelli G. (2020). Polyamines and kynurenines at the intersection of immune modulation. Trends Immunol..

[48] Biernacki T., Sandi D., Bencsik K., Vécsei L. (2020). Kynurenines in the pathogenesis of multiple sclerosis: Therapeutic perspectives. Cells.

[49] Huang Y.S., Ogbechi J., Clanchy F.I., Williams R.O., Stone T.W. (2020). IDO and kynurenine metabolites in peripheral and CNS disorders. Front. Immunol..

[50] Zhai L., Bell A., Ladomersky E., Lauing K.L., Bollu L., Sosman J.A., Zhang B., Wu J.D., Miller S.D., Meeks J.J. (2020). Immunosuppressive IDO in cancer: Mechanisms of action, animal models, and targeting strategies. Front. Immunol..

[51] Lee J.H., Wood T.K., Lee J. (2015). Roles of indole as an interspecies and interkingdom signaling molecule. Trends Microbiol..

[52] Sardar P., Kempken F. (2018). Characterization of indole-3-pyruvic acid pathway-mediated biosynthesis of auxin in Neurospora crassa. PLoS ONE.

[53] González B., Vázquez J., Morcillo-Parra M.Á., Mas A., Torija M.J., Beltran G. (2018). The production of aromatic alcohols in non-Saccharomyces wine yeast is modulated by nutrient availability. Food Microbiol..

[54] Cornford E.M., Bocash W.D., Braun L.D., Crane P.D., Oldendorf W.H., MacInnis A.J. (1979). Rapid distribution of tryptophol (3-indole ethanol) to the brain and other tissues. J. Clin. Investig..

[55] Feldstein A., Chang F.H., Kucharski J.M. (1970). Tryptophol, 5-hydroxytryptophol and 5-methoxytryptophol induced sleep in mice. Life Sci..

[56] Laćan G., Magnus V., Jeričević B., Kunst L., Iskrić S. (1984). Formation of tryptophol galactoside and an unknown tryptophol ester in Euglena gracilis. Plant Physiol..

[57] Hofsteenge J., Müller D.R., de Beer T., Löffler A., Richter W.J., Vliegenthart J.F. (1994). New type of linkage between a carbohydrate and a protein: C-glycosylation of a specific tryptophan residue in human RNase Us. Biochemistry.

[58] Gutsche B., Grun C., Scheutzow D., Herderich M. (1999). Tryptophan glycoconjugates in food and human urine. Biochem. J..

[59] Palmieri A., Petrini M. (2019). Tryptophol and derivatives: Natural occurrence and applications to the synthesis of bioactive compounds. Nat. Prod. Rep..

[60] Ray P.D., Foster D.O., Lardy H.A. (1966). Paths of carbon in gluconeogenesis and lipogenesis. IV. Inhibition by L-tryptophan of hepatic gluconeogenesis at the level of phosphoenolpyruvate formation. J. Biol. Chem..

[61] Smith S.A., Elliott K.R.F., Pogson C.I. (1978). Differential effects of tryptophan on glucose synthesis in rats and guinea pigs. Biochem. J..

[62] Badawy A.A. (2017). Kynurenine pathway of tryptophan metabolism: Regulatory and functional aspects. Int. J. Tryptophan Res..

[63] Davidson V.L., Liu A. (2012). Tryptophan tryptophylquinone biosynthesis: A radical approach to posttranslational modification. Biochim. Biophys. Acta.

[64] Davidson V.L. (2018). Protein-derived cofactors revisited: Empowering amino acid residues with new functions. Biochemistry.

[65] McIntire W.S., Wemmer D.E., Chistoserdov A., Lidstrom M.E. (1991). A new cofactor in a prokaryotic enzyme: Tryptophan tryptophylquinone as the redox prosthetic group in methylamine dehydrogenase. Science.

[66] Davidson V.L., Wilmot C.M. (2013). Posttranslational biosynthesis of the protein-derived cofactor tryptophan tryptophylquinone. Annu. Rev. Biochem..

[67] Davidson V.L. (2001). Pyrroloquinoline quinone (PQQ) from methanol dehydrogenase and tryptophan tryptophylquinone (TTQ) from methylamine dehydrogenase. Adv. Protein. Chem..

[68] Datta S., Mori Y., Takagi K., Kawaguchi K., Chen Z.W., Okajima T., Kuroda S., Ikeda T., Kano K., Tanizawa K. (2001). Structure of a quinohemoprotein amine dehydrogenase with an uncommon redox cofactor and highly unusual crosslinking. Proc. Natl. Acad. Sci. USA.

[69] Campillo-Brocal J.C., Chacon-Verdu M.D., Lucas-Elio P., Sanchez-Amat A. (2015). Distribution in microbial genomes of genes similar to lodA and goxA which encode a novel family of quinoproteins with amino acid oxidase activity. BMC Genom..

[70] Campillo-Brocal J.C., Lucas-Elio P., Sanchez-Amat A. (2015). Distribution in different amino acid oxidases with FAD or a quinone as cofactor and their role as antimicrobial proteins in marine bacteria. Mar. Drugs.

[71] Schlamadinger D.E., Gable J.E., Kim J.E. (2009). Hydrogen bonding and solvent polarity markers in the UV resonance Raman spectrum of tryptophan: Application to membrane proteins. J. Phys. Chem. B.

[72] Thomas Jr G.J. (2002). New structural insights from Raman spectroscopy of proteins and their assemblies. Biopolymers.

[73] Takeuchi H. (2011). UV Raman markers for structural analysis of aromatic side chains in proteins. Anal. Sci..

[74] Weiler-Feilchenfeld H., Pullman A., Berthod H., Giessner-Prettre C. (1970). Experimental and quantum-chemical studies of the dipole moments of quinoline and indole. J. Mol. Struct..

[75] Galgonek J., Vymetal J., Jakubec D., Vondrášek J. (2017). Amino Acid Interaction (INTAA) web server. Nucleic Acids Res..

[76] Barik S. (2019). Protein tetratricopeptide repeat and the companion non-tetratricopeptide repeat helices: Bioinformatic analysis of interhelical interactions. Bioinform. Biol. Insights.

[77] Barik S. (2020). The nature and arrangement of pentatricopeptide domains and the linker sequences between them. Bioinform. Biol. Insights.

[78] Small I.D., Peeters N. (2000). The PPR motif–a TPR-related motif prevalent in plant organellar proteins. Trends Biochem. Sci..

[79] Barkan A., Small I. (2014). Pentatricopeptide repeat proteins in plants. Annu. Rev. Plant. Biol..

[80] Main E.R.G., Xiong Y., Cocco M.J., D’Andrea L., Regan L. (2003). Design of stable alpha-helical arrays from an idealized TPR motif. Structure.

[81] Gallivan J.P., Dougherty D.A. (1999). Cation-pi interactions in structural biology. Proc. Natl. Acad. Sci. USA.

[82] Olson C.A., Shi Z., Kallenbach N.R. (2001). Polar interactions with aromatic side chains in alpha-helical peptides: Ch...O H-bonding and cation-pi interactions. J. Am. Chem. Soc..

[83] Hait S., Mallik S., Basu S., Kundu S. (2020). Finding the generalized molecular principles of protein thermal stability. Proteins.

[84] The UniProt Consortium (2019). UniProt: A worldwide hub of protein knowledge. Nucleic Acids Res..

[85] Jayasinghe S., Hristova K., White S.H. (2001). MPtopo: A database of membrane protein topology. Protein Sci..

[86] Granseth E., von Heijne G., Elofsson A. (2005). A study of the membrane-water interface region of membrane proteins. J. Mol. Biol..

[87] Sanchez K.M., Gable J.E., Schlamadinger D.E., Kim J.E. (2008). Effects of tryptophan microenvironment, soluble domain, and vesicle size on the thermodynamics of membrane protein folding: Lessons from the transmembrane protein OmpA. Biochemistry.

[88] Sanchez K.M., Kang G., Wu B., Kim J.E. (2011). Tryptophan-lipid interactions in membrane protein folding probed by ultraviolet resonance Raman and fluorescence spectroscopy. Biophys. J..

[89] Persson S., Killian J.A., Lindblom G. (1998). Molecular ordering of interfacially localized tryptophan analogs in ester- and ether-lipid bilayers studied by H-2-NMR. Biophys. J..

[90] van der Wel P.C.A., Reed N.D., Koeppe R.E. (2007). Orientation and motion of tryptophan interfacial anchors in membrane-spanning peptides. Biochemistry.

[91] Shafaat H.S., Sanchez K.M., Kim J.E. (2008). Ultraviolet resonance Raman spectroscopy of a membrane-bound beta-sheet peptide as a model for membrane protein folding. J. Raman Spectrosc..

[92] Sun H., Greathouse D.V., Koeppe R.E. (2008). The preference of tryptophan for membrane interfaces: Insights from N-methylation of tryptophans in gramicidin channels. J. Biol. Chem..

[93] Haney E.F., Straus S.K., Hancock R.E.W. (2019). Reassessing the host defense peptide landscape. Front. Chem..

[94] Hong H., Park S., Jimenez R.H.F., Rinehard D., Tamm L.K. (2007). Role of aromatic side chains in the folding and thermodynamic stability of integral membrane proteins. J. Am. Chem. Soc..

[95] Bhattacharjya S., Straus S.K. (2020). Design, engineering and discovery of novel α-helical and β-boomerang antimicrobial peptides against drug resistant bacteria. Int. J. Mol. Sci..

[96] Necelis M.R., Santiago-Ortiz L.E., Caputo G.A. (2020). Investigation of the role of aromatic residues in the antimicrobial peptide BuCATHL4B. Protein Pept. Lett..

[97] Liu Y., Shi J., Tong Z., Jia Y., Yang K., Wang Z. (2020). Potent broad-spectrum antibacterial activity of amphiphilic peptides against multidrug-resistant bacteria. Microorganisms.

[98] Kelkar D.A., Chattopadhyay A. (2007). The gramicidin ion channel: A model membrane protein. Biochim. Biophys. Acta..

[99] Becker M.D., Greathouse D.V., Koeppe R.E., Andersen O.S. (1991). Amino acid sequence modulation of gramicidin channel function: Effects of tryptophan-to-phenylalanine substitutions on the single-channel conductance and duration. Biochemistry.

[100] Salom D., Pérez-Payá E., Pascal J., Abad C. (1998). Environment- and sequence-dependent modulation of the double-stranded to single-stranded conformational transition of gramicidin A in membranes. Biochemistry.

[101] Cotten M., Xu F., Cross T.A. (1997). Protein stability and conformational rearrangements in lipid bilayers: Linear gramicidin, a model system. Biophys. J..

[102] Chaudhuri A., Haldar S., Sun H., Koeppe II R.R., Chattopadhyay A. (2014). Importance of indole N-single bond-H hydrogen bonding in the organization and dynamics of gramicidin channels. Biochim. Biophys. Acta..

[103] Scheiner S., Kar T., Pattanayak J. (2002). Comparison of various types of hydrogen bonds involving aromatic amino acids. J. Am. Chem. Soc..

[104] Neidigh J.W., Fesinmeyer R.M., Prickett K.S., Andersen N.H. (2001). Exendin-4 and glucagon-like-peptide-1: NMR structural comparisons in the solution and micelle-associated states. Biochemistry.

[105] Neidigh J.W., Fesinmeyer R.M., Andersen N.H. (2002). Designing a 20-residue protein. Nat. Struct. Biol..

[106] Qiu L., Pabit S.A., Roitberg A.E., Hagen S.J. (2002). Smaller and faster: The 20-residue Trp-cage protein folds in 4 micros. J. Am. Chem. Soc..

[107] Byrne A., Williams D.V., Barua B., Hagen S.J., Kier B.L., Andersen N.H. (2014). Folding dynamics and pathways of the trp-cage miniproteins. Biochemistry.

[108] Barua B., Lin J.C., Williams V.D., Kummler P., Neidigh J.W., Andersen N.H. (2008). The Trp-cage: Optimizing the stability of a globular miniprotein. Protein Eng. Des. Sel..

[109] Sobash P.T., Kolhe R., Karim N.A., Guddati A.K., Jillella A., Kota V. (2020). Role of indoleamine 2,3-dioxygenase in acute myeloid leukemia. Future Oncol..

[110] Blasco H., Bessy C., Plantier L., Lefevre A., Piver E., Bernard L., Marlet J., Stefic K., Benz-de Bretagne I., Cannet P. (2020). The specific metabolome profiling of patients infected by SARS-COV-2 supports the key role of tryptophan-nicotinamide pathway and cytosine metabolism. Sci. Rep..

[111] Ma N., He T., Johnston L.J., Ma X. (2020). Host-microbiome interactions: The aryl hydrocarbon receptor as a critical node in tryptophan metabolites to brain signaling. Gut Microbes.

[112] Hényková E., Vránová H.P., Amakorová P., Pospíšil T., Žukauskaitė A., Vlčková M., Urbánek L., Novák O., Mareš J., Kaňovský P. (2016). Stable isotope dilution ultra-high performance liquid chromatography-tandem mass spectrometry quantitative profiling of tryptophan-related neuroactive substances in human serum and cerebrospinal fluid. J. Chromatogr. A.

[113] Ozaki Y., Edelstein M.P., Duch D.S. (1988). Induction of indoleamine 2,3-dioxygenase: A mechanism of the antitumor activity of interferon gamma. Proc. Natl. Acad. Sci. USA.

[114] Feng G.S., Taylor M.W. (1989). Interferon gamma-resistant mutants are defective in the induction of indoleamine 2,3-dioxygenase. Proc. Natl. Acad. Sci. USA.

[115] Frumento G., Rotondo R., Tonetti M., Damonte G., Benatti U., Ferrara G.B. (2002). Tryptophan-derived catabolites are responsible for inhibition of T and natural killer cell proliferation induced by indoleamine 2,3-dioxygenase. J. Exp. Med..

[116] Hwu P., Du M.X., Lapointe R., Do M., Taylor M.W., Young H.A. (2000). Indoleamine 2,3-dioxygenase production by human dendritic cells results in the inhibition of T cell proliferation. J. Immunol..

[117] Munn D.H., Sharma M.D., Lee J.R., Jhaver K.J., Johnson T.S., Keskin D.B., Marshall B., Chandler P., Antonia S.J., Burgess R. (2002). Potential regulatory function of human dendritic cells expressing indoleamine 2,3-dioxygenase. Science.

[118] Orabona C., Grohmann U. (2011). Indoleamine 2,3-dioxygenase and regulatory function: Tryptophan starvation and beyond. Methods Mol. Biol..

[119] Dai W., Pan H., Kwok O., Dubey J.P. (1994). Human indoleamine 2,3-dioxygenase inhibits *Toxoplasma gondii* growth in fibroblast cells. J. Interferon Res..

[120] Mehraj V., Routy J.P. (2015). Tryptophan catabolism in chronic viral infections: Handling uninvited guests. Int. J. Tryptophan Res..

[121] Mellor A.L., Munn D.H. (2003). Tryptophan catabolism and regulation of adaptive immunity. J. Immunol..

[122] Adams O., Besken K., Oberdörfer C., MacKenzie C.R., Rüssing D., Däubener W. (2004). Inhibition of human herpes simplex virus type 2 by interferon gamma and tumor necrosis factor alpha is mediated by indoleamine 2,3-dioxygenase. Microbes Infect..

[123] Huang L., Li L., Klonowski K.D., Tompkins S.M., Tripp R.A., Mellor A.L. (2013). Induction and role of indoleamine 2,3 dioxygenase in mouse models of influenza A virus infection. PLoS ONE.

[124] Mao R., Zhang J., Jiang D., Cai D., Levy J.M., Cuconati A., Block T.M., Guo J.T., Guo H. (2011). Indoleamine 2,3-dioxygenase mediates the antiviral effect of gamma interferon against hepatitis B virus in human hepatocyte-derived cells. J. Virol..

[125] Obojes K., Andres O., Kim K.S., Däubener W., Schneider-Schaulies J. (2005). Indoleamine 2,3-dioxygenase mediates cell type-specific anti-measles virus activity of gamma interferon. J. Virol..

[126] Sage L.K., Fox J.M., Mellor A.L., Tompkins S.M., Tripp R.A. (2014). Indoleamine 2,3-dioxygenase (IDO) activity during the primary immune response to influenza infection modifies the memory T cell response to influenza challenge. Viral Immunol..

[127] Rabbani M.A.G., Ribaudo M., Guo J.-T., Barik S. (2016). Identification of interferon-stimulated gene proteins that inhibit human parainfluenza virus type 3. J. Virol..

[128] Schmidt S.V., Schultze J.L. (2014). New insights into IDO biology in bacterial and viral infections. Front. Immunol..

[129] Bitko V., Musiyenko A., Shulyayeva O., Barik S. (2005). Inhibition of respiratory viruses by nasally administered siRNA. Nat. Med..

[130] Villenave R., Shields M.D., Power U.F. (2013). Respiratory syncytial virus interaction with human airway epithelium. Trends Microbiol..

